# Violence against Women: An Exploration of the Physical and Mental Health Trends among Immigrant and Refugee Women in Canada

**DOI:** 10.1155/2012/434592

**Published:** 2012-05-20

**Authors:** Sepali Guruge, Brenda Roche, Cristina Catallo

**Affiliations:** ^1^Daphne Cockwell School of Nursing, Ryerson University, 350 Victoria Street, Toronto, ON, Canada M5B 2K3; ^2^Wellesley Institute, 10 Alcorn Avenue, Toronto, ON, Canada M4V 3B2

## Abstract

Violence against women is a serious health and social problem for women worldwide. Researchers have investigated the broad physical and mental health consequences of violence against women but few have focused on immigrant and refugee women. We assessed the history of violence and the impairment of physical and mental health among 60 women participants from the Iranian and Sri Lankan Tamil communities in Toronto, Canada. Our survey findings revealed that the participants had experienced various types of violence throughout their lifespan, with psychological abuse by a spouse/partner occurring most frequently in the past 12 months. Commonly reported types of abuse included insulting, criticizing, and intimidation by partner (psychological abuse); slapping, hitting, and shoving (physical abuse); and forced sexual intercourse and sexually degrading acts (sexual abuse) by a partner/spouse. We found that a substantial proportion of the participants also had experienced physical and mental health impairment, which could be a result of the various types of violence they had experienced throughout their lifespan. Research and practice implications are provided.

## 1. Introduction

Violence against women is a global phenomenon and involves a spectrum of physical, sexual, and psychological acts of control, threat, aggression, abuse, and assault. Violence against women takes many forms, such as female infanticide, (girl) child abuse, incest, rape, sexual harassment, intimate partner violence (IPV), and abuse and neglect of older women. Although health science researchers are increasingly focusing on violence against women at local, national, and international levels, few have investigated violence against women across the lifespan (i.e., violence experienced prior to, during, and after migration) in various immigrant communities. Given the increase in international migration over the past few decades, there is a greater need to examine the experiences and effects of violence among immigrant and refugee women. To date, no published studies have focused on the experiences of violence throughout the lifespan and the physical and mental health consequences among immigrant and refugee women in Canada, even though more than 250,000 immigrants and refugees come to Canada annually, with a significant proportion arriving from the Middle East and Asia [1]. This paper presents the findings of a pilot study that examined women's experience of violence throughout the lifespan and the presence of physical and mental health symptoms in a sample of Iranian and Sri Lankan Tamil immigrants and refugee women in Canada.

## 2. Background

Studies have consistently demonstrated that women and girls worldwide experience various forms of violence [2, 3] by close and extended family members, neighbors, acquaintances, and by men in positions of power and authority such as police and soldiers. Women who are displaced within their own country and those who seek political asylum elsewhere often live in isolated or temporary accommodation or institutional settings and may be particularly vulnerable to such violence, with few safe options [4]. Women who leave an abusive spouse may face additional social and legal consequences if they reveal their experiences or seek help, such as exclusion from their community or problems related to immigration and legal status [5].

 Prevalence research about violence against women, on national and international levels, generally involves methodological challenges. Definitional issues are one problem: what constitutes violence in one setting may not be interpreted as such in another. Beliefs about gender roles and norms, and structural dimensions that may support violence against women (such as patriarchal systems and unequal power relations) vary cross-culturally. These inconsistencies may lead to underreporting of violence in some settings. Moreover, barriers to reporting (e.g., taboo, stigma) may significantly impede accurate collection of epidemiological data.

 Nonetheless, previous research [6, 7] has demonstrated a pervasive trend of violence against women, both cross-culturally and cross-nationally. Violence against women has many physical and mental health consequences that can last long after the violence has ended, resulting in serious public health implications [6–9]. Physical health consequences may include injuries, chronic pain, fibromyalgia, headaches, gastrointestinal disorders, and sexually transmitted diseases [8, 10]. Psychological health consequences may include depression, anxiety, trauma symptoms and extreme stress reactions such as nightmares and sleep disorders, and suicidal ideation [11–13]. During the last few decades, many studies (e.g., [14–21]) have investigated the immediate and long-term health consequences of violence against women and girls, but few have focused on the experiences of violence against immigrant and refugee women and the related health consequences. The studies that do focus on these experiences generally examine premigration violence, postmigration violence, or violence encountered during border-crossing in isolation, rather than all three contexts in one study.

 Women comprise approximately half of all international migrants [22]. Similar patterns are observed in Canada: approximately half of all asylum-seekers, refugees, and immigrants to Canada are women [1]. Researchers have recently begun to focus more on health issues such as mental health and trauma especially among refugees and asylum-seekers [23–25]. Premigration conflict and forced migration can result in various physical, mental, economic, and social consequences. Some of these are related to routine difficulties associated with poverty and deprivation, while others are related to more extreme problems such as war-related injuries, torture, and sexual violence [26, 27]. Immigrants may face a less chaotic journey as they travel to and settle in a new country, but they may also have left their home country because of political, social, and economic difficulties, which can have physical and mental health consequences.

 Migration to a new country can contribute to an increased risk of intimate partner violence, which is one form of violence against women [28]. Emerging evidence suggests that the complex processes involved in migration and (re)settlement, which may include shifts in power dynamics, may leave women especially vulnerable to partner violence [29, 30]. In the postmigration and (re)settlement context, the unanticipated social and economic barriers such as social isolation and un/underemployment encountered by women and their spouses contribute to stress, family conflict, and partner violence [30]. These barriers continue to affect couples even after the initial (re)settlement period [31].

Despite the substantial contribution of health science research to the field of violence and health, there are considerable gaps in our knowledge. For example, there is a significant gap in health science research on violence against immigrant and refugee women, particularly their exposure to different forms of violence across their lifespan, and the resulting physical and mental health problems. As noted earlier, most previous studies of violence against immigrant and/or refugee women have focused exclusively on the postmigration context or the premigration context. Together, information about violence before, during, and after migration can provide important insights to guide intervention efforts for immigrant and refugee women [32].

## 3. Study Purpose

The goal of our pilot study was to describe the trends in violence throughout the lifespans of women who came to Canada as immigrants or refugees and the resulting physical and mental health symptom patterns. We examined the topic in the Iranian and Sri Lankan Tamil communities in Toronto.

## 4. Communities of Focus

### 4.1. Migration History of the Iranian Community in Toronto

Iranians are one of the fast-growing and relatively new communities in Canada. Census records indicate a growth rate of 147% from 1996 to 2006 for this population [33]. The first real wave of Iranian immigrants to Canada arrived in the 1970s, when the number of immigrants increased from 100 to 600 per year by 1978 [34]. Following the Iranian Islamic revolution in 1978, the rate increased to several thousand per year, which continued throughout the Iran/Iraq war and into the 1990s [35]. Over the period of 1978–1990, the majority came to Canada for political reasons. In the period after 1990, most came to Canada for economic reasons [35]. Beyond Canada, annually, more than 100,000 educated Iranian professionals have also immigrated to Australia, United Arab Emirates, and Eastern Europe [36]. In Canada, Toronto has the largest concentration of Iranians [33].

### 4.2. Migration History of the Sri Lankan Tamil Community in Toronto

As a result of the civil war that ravaged Sri Lanka for 25 years, since 1983, almost 60,000 civilians have died, thousands of people have disappeared, and almost one million people have been internally displaced [37]. Many others have sought refugee status in countries like India, Norway, Germany, the UK, the USA, and Canada [38, 39]. Canada is the home to the largest Sri Lankan Tamil community outside Sri Lanka. The majority of the Tamils who came to Canada have settled in major cities. In Toronto, the Sri Lankan Tamil community is estimated at more than 300,000, of which the majority arrived within the past 25 years. Before that, the number of Tamils in Toronto would have been in the low hundreds, and for the most part they would have been well-educated, professional, middle- and upper-class Tamils [40]. Post-1983 Tamil immigrants to Canada have come from a broad cross section of the Tamil population in Sri Lanka [37].

## 5. Methods

### 5.1. Design

This pilot study involved a cross-sectional survey of immigrant and refugee women from the Iranian and Sri Lankan Tamil communities in Toronto, Canada. It was based on an European study of women refugees and asylum-seekers in three countries: Scotland, Belgium, and Italy [41]. Researchers from the London School of Hygiene and Tropical Medicine, working with the Scottish Refugee Council and service providers working with refugees in Belgium and Italy, developed the survey for use in assessing women's experience of violence, physical and mental health, and the asylum process [42]. We used the survey to investigate women's experiences of violence throughout the migration process, and the presence of physical and mental health impairments in a Canadian sample. The physical health symptomatology sub scale was adapted from previous scales investigating physical health sequelae of violence amongst women who had experienced forced migration and/or human trafficking. The survey's mental health symptomatology subscale was derived from subscales of the Brief Symptom Inventory (BSI) [43] and the Harvard Trauma Questionnaire (HTQ) (Harvard Program in Refugee Health http://hprt-cambridge.org/). While the survey utilizes other instruments that have undergone psychometric assessment, and its current form has been screened by experts for face validity, further statistical assessments of its reliability and validity have not been performed.

### 5.2. Setting, Sample, and Sampling

A convenience sample of participants was recruited through key contacts within the Iranian and Sri Lankan communities. This strategy was useful in obtaining community-based samples that are not affiliated with clinical or social services settings. An advantage of this method lies in its ability to identify members of communities that may be hard to reach [44]. Inclusion criteria were that women had immigrated to Canada within the last 15 years from one of the two communities and were 18 years or older and living in the Greater Toronto Area at the time of the study. Because we were not aware of any studies to date exploring violence using a sample of Iranian and Sri Lankan women, we did not carry out a power analysis and sample size calculation. Instead, we used the general principles for sample size recommended for pilot studies, that is, 30 participants per group, for a total of 60 participants [45].

### 5.3. Data Collection

Under the supervision of the Principal Investigator (Guruge) and the Co-principal Investigator (Roche), surveys were conducted by trained research assistants (RAs) who were immigrants themselves, had prior experience in immigration research, and were fluent in the languages spoken by participants. The RAs carried out surveys in each participant's own language and in places that were convenient for participants, and each survey took approximately 1.5 to 2 hours.

Before administering the survey, each participant read and signed a consent form written in her own language. The RAs informed each participant of her right to (a) refuse to participate in the study, (b) refuse to answer any specific questions, or request to switch to a different set of questions, or (c) to end the survey altogether at any time. An honorarium of $30 was provided to each participant to cover the costs (travel, time, child/grandchild care) incurred by participation. Participants were offered a list of resources and services available in their first language and in English. The study was approved by the Ryerson University Research Ethics Board.

### 5.4. Data Entry and Analysis

Analyses were conducted using SPSS version 19. Data were entered by a trained RA, and a member of our research team (Catallo) conducted a review for accuracy. In keeping with our goal of elucidating the trends in violence and physical and mental health symptoms among our two sample groups, we carried out descriptive statistics. To test for possible relationships between violence and physical and mental health symptoms, we computed the total score for violence items and the total scores for the physical and mental health items, which were then analyzed for correlation (Pearson r). The mean and standard deviation for the total scores were also calculated.

## 6. Results

This section presents our findings in the following order: (1) demographics, (2) experiences of violence, (3) physical health, (4) mental health, and (5) relationship(s) between violence and physical and mental health.

### 6.1. Demographics

As can be seen in Table 1, the majority of women from Iran were aged 31–40 years. In contrast, the ages of Sri Lankan Tamil women were evenly distributed across age groups; overall, they were older than the Iranian group. Both groups included women who were educated, highly literate in their first language, and comfortable speaking in English. Almost 75% (*n* = 22) of the Iranian sample had college/university and/or postgraduate education, and a similar percentage (i.e., 77%; *n* = 23) had been employed in their country of origin. Half of the Tamil sample had college/university and/or postgraduate education. A smaller percentage (27%; *n* = 8) of Sri Lankan Tamil women had been employed prior to migration. The latter rates may be explained by the education and employment disruption experienced by Tamils in the war-torn Sri Lanka. Given the lengthy civil war that ravaged the country, it is not surprising that 60% (*n* = 18) of Sri Lankan Tamil women reported having stayed at a transit location such as a refugee camp or a detention center. In both groups, 83% (*n* = 23) women were married and more than 75% had children. Both groups included participants with a variety of immigration statuses, including those who came to Canada as landed immigrants, family members who were sponsored as immigrants, and individuals and families who came as refugees.

### 6.2. Experiences of Violence

Participants reported experiencing various forms of violence, including as a child witnessing physical or sexual violence (Table 2); experience of physical violence before the age of 15 (Table 3); experience of sexual violence before the age of 15 (Table 4); experience of physical violence after the age of 15 by someone other than an intimate partner (Table 5); experience of sexual violence after the age of 15 by someone other than an intimate partner (Table 6); and experience of intimate partner violence (Tables 7 and 8). The data pertaining to each form of violence are discussed next.

Of the Iranian participants, 20% (*n* = 6) had witnessed physical or sexual violence as a child. We were surprised that only one Sri Lankan Tamil woman reported witnessing violence as a child (and that 53% (*n* = 16) reported witnessing no violence as a child) given that almost half the samples were aged 15 or younger during the civil war, and that war-like situations generally tend to increase violence. Overall, 67% (*n* = 20) of the Iranian group and the 43% (*n* = 13) of the Sri Lankan Tamil group selected “no response.” While none of the items for the entire instrument had missing data, the selection of “no response” could have been deliberate and in keeping with the groups' social norms, that is, that disclosing violence is not socially acceptable. It is also possible that women were unsure whether what they witnessed as a child was physical or sexual violence.

Of Iranian participants, 23% (*n* = 7) reported experiencing physical violence by a family member. None of the Sri Lankan Tamil women reported experiencing physical violence before the age of 15 years. The latter rates may be underreported: while the rates of physical punishment of children in Sri Lanka have decreased substantially, they were higher in previous decades. The considerable number of Iranian women (i.e., *n* = 3–10; 10–35%) who answered “no response” also requires more investigation; they may also have been uncomfortable acknowledging such experiences.

Only a small portion of the sample (*n* = 4, 13% in the Iranian sample; and *n* = 3, 10% in the Sri Lankan Tamil sample) acknowledged experiencing sexual violence before the age of 15 years. The “no response” rate for sexual violence before the age of 15 years was 7–17% (*n* = 2–5) in the Iranian community and 3–10% (*n* = 1–3) in the Sri Lankan Tamil community. This appears to be the same group of participants who consistently reported “no response” except for the last item, where some of the women who had answered “no response” to previous items did acknowledge the violence they had experienced by a human trafficker. Given the civil war context in Sri Lanka over the last 25 years, it is possible that the rates are underreported in the Sri Lankan sample. Violence against girls and women (and sexual violence in particular) is known to increase in the context of war [46–48].

Approximately 30% (*n* = 9) of the participants in the Iranian sample and about 36% (*n* = 11) of the Sri Lankan sample acknowledged having experienced physical violence since the age of 15 years. “No response” rates ranged from 20 to 70% (*n* = 6–21) in the Iranian group and from 3 to 33% (*n* = 1–10) in the Sri Lankan Tamil group. The questions strived to capture a spectrum of experiences of violence in the lives of women: for example, violence perpetrated by a family member to violence in the context of conflict or political unrest, and violence related to participants' experience of being a refugee. (Spouses/partners were not included as a category in this question. Information on violence by a partner/spouse is explored separately, in Tables 7 and 8.) We can speculate that experiences of violence at the hands of strangers (such as government officials, rebels, community officials, and refugee centre staff) hold particular meanings to victims. For this reason, it is possible that some participants chose “no response” rather than denying their experience altogether.

No women in either group reported experiencing sexual violence since the age of 15 years. In these communities, experiences of sexual violence can lead to considerable stigma and embarrassment, which may limit not only a victim's opportunities for marriage but also those of her siblings and/or (future) children. For this reason, it is possible that at least some participants may have chosen to answer “no.”

Altogether, 43% (*n* = 13) participants in the Iranian group and 63% (*n* = 19) participants in the Sri Lankan Tamil group reported ever having experienced intimate partner violence.  Table 8 provides a breakdown of the report of violence based on the timeline of the experience.

In the Iranian group, a maximum of 30% (*n* = 9) reported psychological violence, 7% (*n* = 2) physical violence, and 7% (*n* = 2) sexual violence in the past 12 months. In comparison, a maximum of 27% (*n* = 8) reported psychological violence, 10% (*n* = 3) physical violence, and 17% (*n* = 5) sexual violence before 12 months. Approximately 37% (*n* = 11) reported psychological violence, 13% (*n* = 4) physical violence, and 17% (*n* = 5) sexual violence in the home country or during transit took place PRIOR to the past 12 months.

In the Sri Lankan Tamil group, 30% (*n* = 9) reported psychological violence, 13% (*n* = 4) reported physical violence, and none reported sexual violence, for the past 12 months. In comparison, 10% (*n* = 3) reported experiencing psychological violence, 10% (*n* = 3) reported physical violence, and none reported sexual violence before 12 months. Of this group, 27% (*n* = 8) reported psychological violence and 3% (*n* = 1) experienced physical violence in the home country or during transit. None reported sexual violence during transit or in their home country.

 These results may be related to the general perception that physical and sexual violence is considered more severe than psychological violence, or that sexual violence, in general, is not to be discussed with “outsiders” [49].

### 6.3. Physical Health

Table 9 provides overall ratings of physical health for the two groups. Interestingly, 93% (*n* = 28) of Iranian participants and 60% (*n* = 18) of Sri Lankan Tamil participants reported their overall health in the past four weeks as good to excellent even though 23% (*n* = 7) of Iranian participants and 37% (*n* = 11) of Sri Lankan Tamil participants reported physical problems limiting their activities and approximately 40% (*n* = 12-13) of participants in both groups reported moderate to severe physical pain. Additionally, 57% (*n* = 17) in the Iranian group and 60% (*n* = 18) in the Tamil group reported none to very little physical energy in the past four weeks.

As can be seen in Table 10, both groups reported high rates of headache, trouble remembering things, back pain, and colds, infections, and flu in the past four weeks. All physical symptoms appeared more among Sri Lankan Tamil women than among Iranian women.

### 6.4. Mental Health

In both groups, one-third of participants reported mental health symptoms in all of the 28 measured symptoms during the past seven days (see Table 11). Iranian participants reported higher rates of mental health sequelae in the following areas: feeling detached or withdrawn, inability to feel emotional, inability to remember traumatic events, sudden emotional or physical reaction when reminded of trauma, feeling worthless, feeling hopeless, and feeling tense or keyed up. Sri Lankan Tamil participants reported higher rates of recurrent nightmares, feeling jumpy and startled, feeling scared, trouble sleeping, feeling fearful about things, and spells of terror or panic. One woman in each group had thought about committing suicide during the past seven days. One Iranian participant and three Sri Lankan Tamil participants reported attempting suicide in their lifetime.

### 6.5. Relationships between Violence and Physical and Mental Health

As can be seen in Table 12, the mean prevalence of violence was 16.75 with scores ranging from 0 to 144. The mean physical health symptom prevalence was 3.82 with scores ranging from 0 to 12, and the mean mental health symptom prevalence was 15.12 with scores ranging from 0 to 39.

No statistically significant associations were found between total scores for violence and total scores for physical and mental health symptoms (see Table 13).

### 6.6. Study Limitations

This pilot study included a convenience sample of immigrant and refugee women, which may have unintentionally excluded women with specific histories of violence. While we adhered to the norms of sample size for pilot studies, it is difficult to know whether or not the lack of correlation found between violence total scores and the physical and mental health total scores is due to the small sample size. It is also difficult to know whether the considerable number of “no responses” received was due to errors within the instrument itself or related to the stigma and taboo associated with violence in these communities. Thus, in addition to conducting a psychometric evaluation of this instrument, future studies should consider a sensitivity analysis to assess the factors that may be contributing to the high rates of “no response.” Survey tools used to assess sensitive topics (including those used in our study) involve methodological limitations. For example, participants' perceptions of “sexual violence” witnessed and/or experienced under the age of 15 years are shaped by a complex set of factors. Future research could therefore use a mixed-method design to allow for qualitative assessment of women's experiences. Despite the above-noted limitations, this pilot study provides new and compelling evidence regarding the trends in violence and mental and physical health symptoms among immigrant and refugee women and highlights the pervasiveness of violence throughout their lifespans.

## 7. Discussion

This work contributes to a growing body of work that seeks to examine the impact of violence on women's lives. Participants in both the Iranian and Sri Lankan Tamil groups reported psychological abuse most often, followed by physical and sexual abuse, during the past 12 months. The most commonly reported types of abuse were insulting, criticizing, and intimidation by partner (psychological abuse), slapping, hitting, and shoving (physical abuse), and forced sexual intercourse and being forced to partake in sexually-degrading acts (sexual abuse). These findings are similar to the rates identified in a recent study of Iranian women seeking primary care for exposure to violence from their spouses in the past 12 months [50]; the authors found that psychological abuse was most common, followed by sexual abuse and physical abuse. Similar to our study, the most commonly reported actions were insulting, criticizing, forced sexual intercourse, slapping, and pushing. Our findings are also similar to a recent Canadian qualitative study of Sri Lankan immigrant women who identified physical abuse as including hitting, beating, and throwing objects, and psychological abuse as including controlling behaviors, insulting, and criticizing [51]. Other studies in the Sri Lankan Tamil community (e.g., [49]) also noted that women were hesitant to report sexual violence, in particular. It is worth noting that despite the role of stigma and other barriers, the data indicate considerable rates of IPV, particularly during the most recent phases of their migration history. This speaks to the immediacy of violence in women's lives and the urgent need for further research towards intervention.

 In studies of non-immigrant women, violence against women has been associated with poor physical and mental health outcomes [52–55]. We did not find any significant associations between violence and mental and physical health sequelae among the Iranian and Tamil women we surveyed. Nonetheless there is a strong presence of mental and physical health symptoms among this sample of women, many of whom have acknowledged exposure to multiple forms of physical and psychological violence during their lifetime (before-, during, and after migration). Many of the physical symptoms reported can be associated with psychological distress, such as headaches, difficulties with memory, breathing problems, dizzy spells and fainting. Similarly, the list of mental health symptoms suggest difficulties associated with traumatic stress, such as recurrent nightmares, emotional detachment, hyper vigilance, difficulty concentrating and sleeping. The trends observed in our data echo those observed with women refugees in the original European study [41]. Without additional data on the meaning of these symptoms, and the context surrounding their occurrence, we are limited in our ability to interpret their meaning and/or the relationships of these experiences to violence over the life course. The strong patterns of physical and mental health symptoms for this small sample of women suggest that there may be connections or relationships that are worthy of further investigation.

Moreover, while our sample was small, the results suggest that future research about violence against immigrant and refugee women should investigate a variety of demographic factors and their degree of association. For example, one recent study of Iranian women identified the correlates of violence against women to include an age of 20 years or younger, low income, and unemployment [50]. We found that women who were exposed to violence tended to be relatively older (31–40 years), skilled in speaking English, and currently married with 1–3 children. While our survey did not allow us to investigate associations at this level, a considerable proportion of our participants were highly educated, having completed college, university, and/or postgraduate education. The role of education as a protective factor [50] may warrant closer examination. In particular, additional descriptive data may be helpful in clarifying the complex role that education can play across different life circumstances. Furthermore, situations in countries of origin change over time. For example, early Sri Lankan Tamil immigrants to Canada were generally well-educated professionals, but more recent Sri Lankan immigrants have faced life disruption and limited access to education as a result of the civil war [56]. Thus, future research is needed to clarify the correlates of violence for immigrant and refugee women over time and in different geographical locations.

## 8. Implications

### 8.1. Research Implications

This study identified important aspects of immigrant and refugee women's experiences of violence and the health issues that might emerge as a result. Future research is needed to identify the prevalence, typology, and frequency of violence and the resulting physical and mental health impairment among immigrant and refugee women experiencing violence. Such research will need to draw on alternative methods: probability sampling with the goal of obtaining a more representative sample among immigrant and refugee communities could help verify the consistency of these preliminary findings and clarify important associations between health and exposure to different and multiples forms of violence during a woman's lifetime. The use of qualitative methods in conjunction with quantitative assessments may yield important insights into women's perceptions about different types and severities of violence, and what relationships they see (if any) between their physical and mental health symptoms and incidents of violence over their lifetime.

Individuals who routinely encounter violence may underreport particular experiences, remembering and confirming them only when prompted [57]. In-depth qualitative research may be required to clarify the range and depth of issues raised in this study, which to date have received relatively little attention: women's experiences of violence during transit, women's feelings of safety in the postmigration context, posttraumatic stress disorder due to pre-migration trauma, and the impact of asylum-seeking or immigration and (re)settlement on women's mental and physical health. Future research involving immigrant and refugee communities could also aim to develop a practical tool for use by asylum officials and settlement service providers to encourage early identification of women who may have been exposed to violence. Further research will need to clarify the patterns of violence disclosure and the reliability of these tools.

### 8.2. Practice Implications

Our results suggest that immigrant and refugee women exposed to violence may experience considerable symptoms of posttraumatic stress and depression. This finding has important clinical implications for healthcare providers with regard to screening immigrant and refugee women for mental and physical health symptoms. Clarification of whether or not mental health symptoms are linked with exposure to violence against immigrant women (as they are among non-immigrant women) is needed to ensure appropriate assessment and treatment of immigrant and refugee women who have been exposed to violence.

The health problem patterns we identified among our participants reveal that violence affects women in different ways. For example, our Sri Lankan participants reported more physical symptoms than emotional symptoms. Accordingly, if healthcare professionals assess only for mental health symptoms, they will fail to identify the violence and trauma experienced by some women or certain groups of women. Somatization, where an individual experiences physical health symptoms instead of psychological symptoms, figures prominently in cross-cultural research [58]. Healthcare professionals should therefore ask about violence routinely and take a holistic approach to health and violence that includes physical and mental health and illness. It is also important for settlement service providers to help women early in their immigration and (re)settlement process. Settlement organizations are a critical resource for newcomers and are likely to function as points of first contact for new immigrants as they adjust to life in Canada. These agencies have the potential to operate as system facilitators, linking individuals with appropriate health care and support services. While often overlooked as a resource, such agencies are well positioned to offer timely and nonthreatening preventative services as well as interventions to ensure appropriate referrals for counseling, medical care, and other supports.

## Figures and Tables

**Table 1:** Demographics of study participants.

Demographic characteristics	Iranian group *N* = 30 *n* (%)	Sri Lankan group *N* = 30 *n* (%)
Age		
21–30 years	5 (16.7)	8 (26.7)
31–40 years	17 (56.7)	8 (26.7)
41–50 years	3 (10.0)	8 (26.7)
>50 years	4 (13.3)	6 (20.0)
Education		
Elementary school	1 (3.33)	1 (3.33)
High school	7 (23.3)	8 (26.7)
College/university	20 (66.7)	9 (30.0)
Postgraduate	2 (6.67)	6 (20.0)
Ability to read and write in mother tongue		
Can read but not write	1 (3.33)	1 (3.33)
Can read and write	29 (96.7)	29 (96.7)
Ability to speak in English		
None/poor	4 (13.3)	2 (6.67)
Good	18 (60.0)	17 (56.7)
Very good	8 (26.7)	7 (23.3)
Excellent	0	4 (13.3)
Past employment in home country		
Yes	23 (76.7)	8 (26.7)
No	7 (23.3)	22 (73.3)
Current marital status		
Never married	2 (6.67)	1 (3.33)
Married	25 (83.3)	25 (83.3)
Divorced/separated	2 (6.67)	0
Widowed	1 (3.33)	3 (10.0)
Location of current husband/partner		
Living together in new country	22 (73.3)	25 (83.3)
Living apart in new country	0	1 (3.33)
Living in another country	3 (10.0)	0
No response	5 (16.7)	4 (13.3)
Transit location after leaving home country		
No transit location	26 (86.7)	10 (33.3)
Transit location (e.g., refugee camp, detention centre, other country)	4 (13.3)	18 (60.0)
No response	0	2 (6.67)
Number of children		
No children	5 (16.7)	7 (23.3)
1–3 children	23 (76.7)	20 (66.7)
≥4 children	2 (6.67)	3 (10.0)

**Table 2:** Child witness of physical or sexual violence.

Child witness of physical or sexual violence	Iranian group *N* = 30 *n* (%)	Sri Lankan group *N* = 30 *n* (%)
Yes	6 (20.0)	1 (3.33)
No	4 (13.3)	16 (53.3)
No response	20 (66.7)	13 (43.3)

**Table 3:** Physical violence before the age of 15 years.

	Iranian group *N* = 30				Sri Lankan group *N* = 30			
Physical violence beforethe age of 15 years	Yes In the home country *n* (%)	Yes In Canada *n* (%)	No *n* (%)	No response *n* (%)	Yes In the home country *n* (%)	Yes In Canada *n* (%)	No *n* (%)	No response *n* (%)
By a family member	7 (23.3)	0	20 (66.7)	3 (10.0)	0	1 (3.33)	28 (93.3)	1 (3.33)
By a government official	0	0	20 (66.7)	10 (33.3)	0	0	28 (93.3)	2 (6.67)
By rebels/opposition forces	0	0	20 (66.7)	10 (33.3)	0	0	28 (93.3)	2 (6.67)
Community official	3 (10.0)	0	20 (66.7)	7 (23.3)	0	0	28 (93.3)	2 (6.67)
Refugee centre official	0	0	20 (66.7)	10 (33.3)	0	0	28 (93.3)	2 (6.67)
Human trafficker	0	0	20 (66.7)	10 (33.3)	0	0	28 (93.3)	2 (6.67)

**Table 4:** Sexual violence before the age of 15 years.

	Iranian group *N* = 30				Sri Lankan group *N* = 30			
Sexual violence before the age of 15 years	Yes In the home country *n* (%)	Yes In Canada *n* (%)	No *n* (%)	No response *n* (%)	Yes In the home country *n* (%)	Yes In Canada *n* (%)	No *n* (%)	No response *n* (%)
By a family member	1 (3.33)	0	25 (83.3)	4 (13.3)	1 (3.33)	1 (3.33)	27 (90.0)	1 (3.33)
By a government official	0	0	25 (83.3)	5 (16.7)	0	0	27 (90.0)	3 (10.0)
By rebels/ opposition forces	0	0	25 (83.3)	5 (16.7)	0	0	27 (90.0)	3 (10.0)
Community official	0	0	25 (83.3)	5 (16.7)	0	0	27 (90.0)	3 (10.0)
Refugee centre official	0	0	25 (83.3)	5 (16.7)	0	0	27 (90.0)	3 (10.0)
Human trafficker	3 (10.0)	0	25 (83.3)	2 (6.67)	1 (3.33)	0	27 (90.0)	2 (6.67)

**Table 5:** Physical violence since the age of 15 years.

Physical violence since the age of 15 years	Iranian group *N* = 30				Sri Lankan group *N* = 30			
	Yes In the home country *n* (%)	Yes In Canada *n* (%)	No *n* (%)	No response *n* (%)	Yes In the home country *n* (%)	Yes In Canada *n* (%)	No *n* (%)	No response *n* (%)
By a family member	5 (16.7)	0	21 (70.0)	4 (13.3)	8 (26.7)	1 (3.33)	20 (66.7)	1 (3.33)
By a government official	0	0	21 (70.0)	9 (30.0)	1 (3.33)	0	20 (66.7)	9 (30.0)
By rebels/opposition forces	0	0	21 (70.0)	9 (30.0)	0	0	20 (66.7)	10 (33.3)
Community official	1 (3.33)	0	21 (70.0)	8 (26.7)	2 (6.67)	0	20 (66.7)	8 (26.7)
Refugee centre official	0	0	21 (70.0)	9 (30.0)	0	0	20 (66.7)	10 (33.3)
Other individual	3 (10.0)	0	6 (20.0)	21 (70.0)	0	0	20 (66.7)	10 (33.3)

**Table 6:** Sexual violence since the age of 15 years.

	Iran (*N* = 30)				Sri Lanka (*N* = 30)			
Sexual violence since the age of 15 years	Yes In the home country *n* (%)	Yes In Canada *n* (%)	No *n* (%)	No response *n* (%)	Yes In the home country *n* (%)	Yes In Canada *n* (%)	No *n* (%)	No response *n* (%)
By a family member	0	0	29 (96.7)	1 (3.33)	0	0	28 (93.3)	2 (6.67)
By a government official	0	0	29 (96.7)	1 (3.33)	0	0	28 (93.3)	2 (6.67)
By rebels/opposition forces	0	0	29 (96.7)	1 (3.33)	0	0	28 (93.3)	2 (6.67)
Community official	0	0	29 (96.7)	1 (3.33)	0	0	28 (93.3)	2 (6.67)
Refugee centre official	0	0	29 (96.7)	1 (3.33)	0	0	28 (93.3)	2 (6.67)
Other individual	0	0	29 (96.7)	1 (3.33)	0	0	28 (93.3)	2 (6.67)

**Table 7:** Experience of violence by a partner/spouse during their lifetime.

	Iranian group *N* = 30 *n* (%)	Sri Lankan group *N* = 30 *n* (%)
Report of violence	13 (43.3%)	19 (63.3%)
No Report of violence	17 (56.7%)	11 (36.7%)

**Table 8:** Experience of violence by a partner/spouse in the past 12 months, before 12 months, and in the home country or in transit.

	Iranian group *N* = 30			Sri Lankan group *N* = 30		
Experience of violence	In Canada		In home country or transit *n* (%)	In Canada		In home country or transit *n* (%)
	In the past 12 months *n* (%)	Before 12 months *n* (%)		In the past 12 months *n* (%)	Before 12 months *n* (%)	
Partner insulted and made to feel badly about self	9 (30.0)	8 (26.7)	11 (36.7)	8 (26.7)	3 (10.0)	8 (26.7)
Partner did things to scare and intimidate participant	4 (13.3)	8 (26.7)	9 (30.0)	9 (30.0)	4 (13.3)	5 (16.7)
Partner threatened to hurt participant or someone close	1 (3.33)	2 (6.67)	2 (6.67)	1 (3.33)	0	0
Partner slapped or threw something at participant	2 (6.67)	3 (10.0)	4 (13.3)	4 (13.3)	3 (10.0)	1 (3.33)
Partner pushed or shoved participant	2 (6.67)	3 (10.0)	4 (13.3)	0	0	0
Partner hit participant	2 (6.67)	3 (10.0)	4 (13.3)	0	0	0
Partner kicked, dragged, or beat participant	2 (6.67)	3 (10.0)	4 (13.3)	0	0	0
Partner threatened to use gun/knife/weapon	0	2 (6.67)	2 (6.67)	0	0	0
Partner forced participant to have sexual intercourse	2 (6.67)	5 (16.7)	5 (16.7)	0	0	0
Partner forced participant to do something sexually degrading	1 (3.33)	3 (10.0)	4 (13.3)	0	0	0
Participant had sexual intercourse out of fear	1 (3.33)	3 (10.0)	3 (10.0)	0	0	0
Participant injured badly with pain lasting > 1 day	2 (6.67)	4 (13.3)	5 (16.7)	0	0	0

**Table 9:** Rating of physical health in the past four weeks.

Physical health in the past four weeks	Iranian group *N* = 30 *n* (%)	Sri Lankan group *N* = 30 *n* (%)
Overall rating of health		
Poor	0	3 (10.0)
Fair	2 (6.67)	9 (30.0)
Good	17 (56.7)	14 (46.7)
Very good	6 (20.0)	1 (3.33)
Excellent	5 (16.7)	3 (10.0)
Overall rating of physical problems limiting activities		
None and Very Little	23 (76.7)	19 (63.3)
Moderate to Severe	7 (23.3)	11 (36.7)
Overall rating of physical pain		
None and Very Little	18 (60.0)	18 (60.0)
Moderate to Severe	12 (40.0)	12 (40.0)
Overall rating of physical energy		
None and Very Little	17 (56.7)	18 (60.0)
Good to Excellent	13 (43.3)	12 (40.0)

**Table 10:** Presence of physical symptoms in the past four weeks.

Presences of physical symptoms in the past four weeks	Iranian group *N* = 30 *n* (%)	Sri Lankan group *N* = 30 *n* (%)
Headaches		
Yes	30 (100)	30 (100)
No	0	0
Fainting/losing consciousness		
Yes	0	4 (13.3)
No	30 (100)	26 (86.7)
Dizzy spells		
Yes	3 (10.0)	6 (20.0)
No	27 (90.0)	24 (80.0)
Weight loss		
Yes	2 (6.67)	9 (30.0)
No	28 (93.3)	21 (70.0)
Trouble remembering things		
Yes	10 (33.3)	11 (36.7)
No	20 (66.7)	19 (63.3)
Dental pain		
Yes	4 (13.3)	9 (30.0)
No	26 (86.7)	21 (70.0)
Facial injuries		
Yes	0	1 (3.33)
No	30 (100)	29 (96.7)
Breathing problems		
Yes	1 (3.33)	10 (33.3)
No	29 (96.7)	20 (66.7)
Upset stomach and vomiting		
Yes	5 (16.7)	13 (43.3)
No	25 (83.3)	17 (56.7)
Back pain		
Yes	17 (56.7)	18 (60.0)
No	13 (43.3)	12 (40.0)
Colds, infections, and flu		
Yes	10 (33.3)	15 (50.0)
No	20 (66.7)	15 (50.0)
Painful urination		
Yes	0	1 (3.33)
No	30 (100)	29 (96.7)
Incontinence of bladder and bowel		
Yes	1 (3.33)	11 (36.7)
No	29 (96.7)	19 (63.3)
Unusual vaginal bleeding/discharge		
Yes	1 (3.33)	4 (13.3)
No	29 (96.7)	26 (86.7)
Excess vaginal bleeding/discharge		
Yes	0	1 (3.33)
No	30 (100)	29 (96.7)
Pelvic pain		
Yes	6 (20.0)	16 (53.3)
No	24 (80.0)	14 (46.7)

**Table 11:** Presence of mental health symptoms over the past seven days.

Presence of mental health symptoms over the past seven days	Iranian group *N* = 30 *n* (%)	Sri Lankan group *N* = 30 *n* (%)
Recurrent memories of most hurtful events		
Yes	18 (60.0)	18 (60.0)
No	12 (40.0)	12 (40.0)
Feeling as though frightening event is happening again		
Yes	15 (50.0)	16 (53.3)
No	15 (50.0)	14 (46.7)
Recurrent nightmares		
Yes	9 (30.0)	15 (50.0)
No	21 (70.0)	15 (50.0)
Feeling detached or withdrawn		
Yes	8 (26.7)	7 (23.3)
No	22 (73.3)	23 (76.7)
Inability to feel emotions		
Yes	6 (20.0)	4 (13.3)
No	24 (80.0)	26 (86.7)
Feeling jumpy, easily startled		
Yes	8 (26.7)	19 (63.3)
No	22 (73.3)	11 (36.7)
Difficulty concentrating		
Yes	16 (53.3)	15 (50.0)
No	14 (46.7)	15 (50.0)
Trouble sleeping		
Yes	15 (50.0)	19 (63.3)
No	15 (50.0)	11 (36.7)
Feeling on guard		
Yes	13 (43.3)	14 (46.7)
No	17 (56.7)	16 (53.3)
Feeling irritable or angry outburst		
Yes	19 (63.3)	20 (66.7)
No	11 (36.7)	10 (33.3)
Avoiding activities that trigger memories of trauma		
Yes	17 (56.7)	9 (30.0)
No	13 (43.3)	21 (70.0)
Inability to remember parts of traumatic events		
Yes	5 (16.6)	4 (13.3)
No	25 (83.3)	26 (86.7)
Less interest in daily activities		
Yes	16 (53.3)	10 (33.3)
No	14 (46.7)	20 (66.7)
Feeling there is no future		
Yes	10 (33.3)	4 (13.3)
No	20 (66.7)	26 (86.7)
Avoiding thoughts associated with past traumatic event		
Yes	17 (56.7)	8 (26.7)
No	13 (43.3)	22 (73.3)
Sudden emotional or physical reaction when reminder of trauma		
Yes	20 (66.7)	7 (23.3)
No	10 (33.3)	23 (76.7)
Nervousness or shakiness feelings		
Yes	20 (66.7)	20 (66.7)
No	10 (33.3)	10 (33.3)
Feelings of worthlessness		
Yes	8 (26.7)	4 (13.3)
No	22 (73.3)	26 (86.7)
Suddenly scared		
Yes	7 (23.3)	16 (53.3)
No	23 (76.7)	14 (46.7)
Feeling lonely		
Yes	17 (56.7)	13 (43.3)
No	13 (43.3)	17 (56.7)
Feel blue/very sad		
Yes	18 (60.0)	13 (43.3)
No	12 (40.0)	17 (56.7)
Feeling no interest in things		
Yes	10 (33.3)	11 (36.7)
No	20 (66.7)	19 (63.3)
Feeling fearful about things		
Yes	11 (36.7)	17 (56.7)
No	19 (63.3)	13 (43.3)
Feeling hopeless about the future		
Yes	9 (30.0)	6 (20.0)
No	21 (70.0)	24 (80.0)
Feeling tense or keyed up		
Yes	18 (60.0)	13 (43.3)
No	12 (40.0)	17 (56.7)
Spells of terror or panic		
Yes	4 (13.3)	13 (43.3)
No	26 (86.7)	17 (56.7)
Feeling restless and could not sit still		
Yes	12 (40.0)	15 (50.0)
No	18 (60.0)	15 (50.0)
Thoughts of committing suicide		
Yes	1 (3.33)	1 (3.33)
No	29 (96.7)	29 (96.7)

**Table 12:** Mean and standard deviations for violence and physical and mental health symptoms.

Total score (Sum of scores for all participants)	*N*	Mean	Standard deviation	Minimum	Maximum
Violence	60	16.75	34.568	0	144
Physical health symptoms	60	3.82	3.000	0	12
Mental health symptoms	60	15.12	10.990	0	39

**Table 13:** Correlations between violence and the physical and mental health symptoms.

Total score (sum of scores for all participants)	Pearson *R* value	Approximate significance
Violence and physical health symptoms	0.140	0.285
Violence and mental health symptoms	0.240	0.064
